# Influence of education on sexual and reproductive health service utilization for persons with disabilities in nationwide Bangladesh: an explanatory sequential mixed-methods study

**DOI:** 10.1186/s12978-022-01352-7

**Published:** 2022-02-19

**Authors:** Katherine Coral Du, Arifa Bente Mohosin, Amina Amin, Md Tanvir Hasan

**Affiliations:** 1 Yale College, Yale University, New Haven, CT USA; 2 BRAC James P Grant School of Public Health, BRAC University, 6th Floor, Medona Tower, 28 Mohakhali Commercial Area, Bir Uttom A K Khandakar Road, Dhaka, 1213 Bangladesh

**Keywords:** Sexual and reproductive health, Maternal health, Persons with disabilities, Antenatal care, Delivery care, Postnatal care, Family planning, Low-income population, Bangladesh

## Abstract

**Background:**

Persons with disabilities comprise more than one billion people in the world, yet they are one of the most discriminated groups and face significant health disparities. Particularly in developing countries, which contain 80% of the entire population with disabilities, these individuals experience major barriers in accessing sexual and reproductive health (SRH) services. Education is an important factor that greatly affects individuals’ SRH service utilization. Hence, we sought to investigate the relationship between education and SRH service utilization for persons with disabilities in Bangladesh.

**Methods:**

Using an explanatory sequential mixed-methods design, a total of 5000 persons with disabilities were surveyed for the quantitative component and 15 mini-ethnographic case studies were conducted with persons with disabilities for the qualitative component. Chi-squared tests and logistic regression analyses were performed on the survey data, while the qualitative interviews were coded and their SRH themes synthesized accordingly.

**Results:**

Our quantitative findings show that education statistically significantly increases persons with disabilities’ SRH service utilization of antenatal care, delivery care, postnatal care, and family planning (*P* < 0.05). Interestingly, for persons with disabilities, primary education shows increased adjusted odds of family planning use but is likely not enough to increase antenatal care, delivery care, or postnatal care use; secondary or post-secondary education may be required to improve utilization of these latter services. Qualitative findings support the association between higher education levels and greater SRH service use. Persons with disabilities of lower educational attainment held misinformation and distrust in SRH services and experienced mistreatment by SRH healthcare providers, discouraging them from seeking future SRH services.

**Conclusions:**

We report that higher formal education level is associated with greater SRH service use for persons with disabilities in Bangladesh. Formally educating persons with disabilities expands their SRH knowledge and familiarity with SRH services, as well as leads to more economic opportunities so they can afford SRH services. Increasing formal education levels for persons with disabilities, paired with integrating comprehensive sexuality education (CSE) in their schools, will likely help close the gap in SRH health disparities for this vulnerable population.

## Plain Language Summary

Out of more than one billion persons with disabilities in the world, 80% of them live in developing countries. Persons with disabilities commonly face discrimination and health disparities, particularly experiencing major barriers in accessing sexual and reproductive health (SRH) services. Education is a key factor that often leads to social and economic empowerment, which positively contributes towards individuals’ SRH service utilization. In this paper, we examined the relationship between education and SRH service utilization for persons with disabilities in Bangladesh. We surveyed persons with disabilities across all of Bangladesh on their utilization of SRH services and conducted mini-ethnographic case studies with selected participants to more deeply understand their SRH issues and SRH service utilization. Our survey findings show that education significantly increases persons with disabilities’ SRH service utilization of antenatal care, delivery care, postnatal care, and family planning in Bangladesh. Interestingly, for persons with disabilities, primary education may only be able to increase family planning use while secondary or post-secondary education may be required to increase antenatal care, delivery care, and postnatal care use. Our qualitative findings support the association between higher education levels and greater SRH service use. Persons with disabilities of higher education prioritized obtaining formal SRH services from qualified health care providers, even when financially constrained, while lower educated participants tended to be misinformed and distrustful of formal SRH services. We recommend helping persons with disabilities attain higher formal education levels and including comprehensive sexuality education (CSE) in their schools, as it likely will reduce SRH health disparities for this vulnerable group.

## Background

In the world, persons with disabilities are one of the most marginalized and disadvantaged groups, suffering from a significant lack of basic human rights and difficulties attaining social acceptance [1, 2]. More than one billion persons live with a disability, or 15% of the world’s population, according to the World Health Organization (WHO) [2]. Out of all persons with disabilities, 80% live in developing countries [3]. Persons with disabilities are commonly discriminated against and challenged with greater social, economic, civil, and health disparities compared to those without disabilities [1–3]. Many of the disparities arise from barriers to accessing services, particularly in disadvantaged communities and under poor living conditions. Persons with disabilities experience exclusion, isolation, abuse, as well as lack of educational and economic opportunities on a daily basis [2].

Education is key for individuals to realize their full potential, and it often leads to social and economic empowerment. Supporting the education of persons with disabilities is especially effective in helping them escape poverty. In a developing country, persons with disabilities who are educated have an estimated rate of wage returns of 19%, even higher than the 10% wage returns for persons without disabilities [4]. Despite the clear benefits of education, access to it is a far-reaching barrier for persons with disabilities. Education completion gaps between individuals with and without disabilities are shown in all age groups and both low-income and high-income countries, although these education gaps are more prominent in poorer countries [2]. UNESCO reports that in developing countries 98% of children with disabilities do not attend school and 99% of girls with disabilities are illiterate [1].

A strong positive correlation between education and health outcomes is well established [5, 6]. Education not only directly improves health, but also indirectly via work and economic conditions, social-psychological resources, and health lifestyle [5, 7, 8]. Overall, there are multiple studies detailing the associations between education and various sexual and reproductive health (SRH) outcomes, but minimal research specifically on SRH outcomes of persons with disabilities [5, 9]. A systematic review described how there is a theoretical basis for education improving SRH, through changing preferences for the timing of marriage and fertility as well as changing individuals’ abilities to achieve their preferences by allowing for higher income or greater access to contraceptives such as condoms [9]. They concluded that though education has been linked to improved SRH outcomes in low and middle-income countries (LMICs), the causal effects are usually null. However, there were small effects between increased grade level on lower fertility and HIV positive status. Individuals who are informed about SRH are more likely to act responsibly and have better SRH outcomes [10–12].

For persons with disabilities, the challenges in maintaining adequate SRH are amplified. Though individuals with disabilities should have equal SRH rights as those without disabilities, society continues to disregard their sexual and reproductive concerns, aspirations, and human rights [10]. Disability causes barriers in access to SRH resources, while the inherent vulnerability of individuals with disabilities makes it easier for their SRH rights to be violated. A systematic review of interventions to promote SRH services for persons with disabilities in LMICs concluded that out of the relatively few studies that rigorously evaluate them, most (15 out of 16) focused on information provision and awareness raising [11]. It asserts that interventions need to extend beyond these and directly address barriers that persons with disabilities face in utilizing SRH services.

Beyond lacking SRH information, it is difficult for persons with disabilities to access SRH services. Persons with disabilities seeking SRH resources identify that negative and disrespectful attitudes by service providers is especially prevalent and hurtful [12, 13]. Other deterrents include long queues at health facilities, distant health facilities, high service costs, physical inaccessibility to health facilities, and perceptions that persons with disabilities ought to be asexual [12, 14]. Overall, due to the additional barriers that persons with disabilities face in accessing SRH services, they have poorer SRH service utilization and outcomes than persons without disabilities [11].

The present study aims to investigate the relationship between education and SRH service utilization for individuals with disabilities in Bangladesh. Because preserving and strengthening one’s SRH seems exceptionally out of reach for persons with disabilities, it is worthwhile to determine which factors may be influential in establishing SRH service use. Thus, the objective of this present study is to explore whether the education level of individuals with disabilities is a major factor that determines their SRH service utilization. Though there are a few studies in Bangladesh and other LMICs on SRH service utilization [15, 16], to our knowledge this paper is the first study that comprehensively explores these topics in relation to education and disability utilizing a mixed-methods research design at the national level.

## Methods

The study adopted an explanatory sequential mixed-methods design, with a quantitative nationwide survey followed by qualitative mini-ethnographic case studies [17]. The STROBE cross-sectional reporting guidelines were used to ensure that the required study components were met [18]. The sample size for the nationwide survey was calculated with an aim to generate representative estimates relevant to sexual and reproductive health issues suffered by persons with disabilities and their healthcare utilization at the national and sub-national (divisional) level of Bangladesh. Bangladesh has eight administrative divisions namely Barisal, Chattogram, Dhaka, Khulna, Mymensingh, Rajshahi, Rangpur, and Sylhet. Within each division, there are urban and rural areas. The lowest administrative units in urban areas are wards whereas the lowest administrative units in rural areas are unions [19]. For the sample size calculation, the divisions were considered as strata and within the divisions wards and unions were considered as clusters or primary sampling units. The minimum sample size calculated for a single stratum (division) was found to be 648 considering an 11% prevalence of healthcare use by persons with disabilities [20], with 95% confidence level, 3% margin of error and a design effect of 1.55. After considering the finite population correction factor and anticipating a 10% non-response, the stratum-specific sample size was calculated to be 720. Thus for the whole country with eight divisions, the sample size was calculated to be 5760 (720 × 8). However, the research team members were able to conduct interviews with 5000 persons with disabilities. This is because many of the participants were not interested to participate in the research and some of them were found to have migrated to other areas during the time of survey data collection. Although the larger study gathered data from 5000 participants, this paper analyzed data of 590 participants for whom antenatal care, delivery care and postnatal care service utilization data were available and 1954 participants for whom family planning service utilization data were available. For the qualitative component, a total of 51 persons with disabilities were recruited for case studies in the larger study. Among them 25 were unmarried and 26 were ever married. The qualitative participants were recruited from the quantitative sample. Of the 26 ever married participants, antenatal care, delivery care, postnatal care, and family planning service use data were available for 15 participants and information of these individuals were analyzed in this paper.

In recruiting participants for the survey, a multi-stage stratified-cluster sampling procedure was employed with two stages of selection. Specifically, 30 clusters (unions in rural areas and wards in urban areas) were selected from each stratum (administrative division of Bangladesh) in the first stage, and then from each cluster 24 persons with disabilities (12 males and 12 females) were selected via systematic random sampling in the second stage. The sampling frame for each cluster was developed by the research team utilizing the national disability database maintained by the Ministry of Social welfare and obtaining the list of persons with disabilities reside in the respective areas maintained by the disabled persons’ organizations (DPOs). Female participants with any form of disability between 10 and 49 years old and male participants with any form of disability between 10 and 59 years old were included as study participants. These age ranges were determined from the reproductive ages of these two groups and feedback from the stakeholders of the government of Bangladesh as well as non-governmental organizations (NGOs) and DPOs that provide SRH services to the people of Bangladesh.

The survey questionnaire covered questions related to demographic characteristics of the participants, type of disability and self-assessment of disability status, SRH knowledge, SRH sufferings, and formal/informal SRH service utilization. The survey questionnaire was pretested, and feedback incorporated into the final survey and consent forms. A total of 48 field research assistants (24 male + 24 female) and 8 field coordinators carried out the survey after two weeks of intensive training and education related to SRH and disability. The survey was conducted between July and November 2019. Approximately 45 min were required to conduct an interview. For participants with severe sensory impairments, intellectual disabilities, or autism spectrum disorder, caregivers were interviewed with the participants present. Survey data were collected with tablets using the SurveyCTO mobile data collection platform. About 5% of the interviews were rechecked with the participants to confirm the validity and reliability of the responses.

The qualitative study participants were purposively chosen from the quantitative sample. The selection criteria for recruiting the qualitative participants were socio-demographic characteristics (e.g., age, gender, ethnicity, marital status, geographical location of the households, wealth status), disability type, experience of violence, SRH sufferings, and SRH service utilization. The research team preferred to conduct interviews directly with the persons with disabilities. However, when the participants had communication difficulties, like speech and hearing impairments or neurodevelopmental disabilities, the team conducted interviews with their caregivers in presence of the participants. The same approach was also adopted for interviewing participants from ethnic minority groups, such as Bandarban, where the team encountered language barriers.

The mini-ethnographic case study approach allowed the research team to contextualize and verify the quantitative findings, as well as form a better understanding about persons with disabilities’ everyday life experiences, their thoughts about relationship, sexuality, and other SRH issues. The participants were visited by the research team on different days and in each visit the team spent 40 to 50 min. Most of the interviews took place in participants’ home or at convenient places identified by the participants. Before starting each interview, the researchers obtained formal consent from the persons with disabilities and their suggested caregivers, both verbally and in writing. The researchers eased into conversation on the first visit with an initial introduction and then discussed socio-demographic characteristics and daily activities. On the second visit, the researchers discussed social stigma and misconceptions surrounding disability, participants’ thoughts around relationship, marriage, and family formation. In subsequent visits the researchers covered sensitive SRH topics including sexual behavior, contraceptive use, sufferings from sexually transmitted diseases (STDs), experience of violence and discrimination, coping strategies, and SRH service utilization. To cultivate an in-depth understanding of each case study, the research team joined in transects walks with the participants. This helped them capture the overall cultural setting and attitudes toward persons with disabilities in that particular community.

Descriptive analyses were performed to summarize the demographic characteristics of survey participants. Participants’ education level was defined as no formal education, attended primary school (1–5 years of formal education), attended secondary school (6–10 years of formal education), or completed post-secondary (> 10 years of formal education). Proportions of utilization for different SRH services versus education level were graphed using Microsoft Excel, version 16. Additionally, bivariate analyses (chi-squared tests) were used to test for relationships between utilization of different SRH services (antenatal care, delivery care, postnatal care, family planning) and education levels of the participants. Multivariable logistic regressions were also performed, including possible confounding variables, to examine associations between utilization of different SRH services and participants’ education levels. The confounding variables were incorporated in the multivariable logistic regression models considering their epidemiological importance. For the logistic regression model, if a participant used the specified SRH service, they were given a score of 1 in that category; otherwise, they were given a score of 0 in that category. All statistical analyses incorporated sampling weights considering the multistage sampling design and were performed with Stata, version 16.

For qualitative analyses, the interview recordings and field notes were transcribed both in Bangla and English. Interview transcripts were coded by SRH themes using Atlas.ti, version 7. Common themes were synthesized and presented as study findings. The qualitative and quantitative findings were merged to draw a comprehensive conclusion on the different ways education influences participants’ SRH service utilization.

## Results

### Characteristics of the survey participants

The demographic characteristics of the 5000 survey participants are presented in Table 1. The number of male and female participants was almost equal. About 30% of the participants were from the age group 10 to 19 years, 23% were between 20 and 29 years old, 23% were between 30 and 39 years old, 15% were between 40 and 49 years old, and 9% were between 50 and 59 years old. Almost half of the participants (49%) did not receive formal education, while about 28% had attended primary school, 17% had attended secondary school, and only 6% had completed post-secondary or above level of education. The majority of the participants (84%) lived in rural areas, while the rest (16%) lived in urban areas at the time of survey data collection. More than one-third (37%) of the survey participants were married, 54% were unmarried, and the left over 9% of the participants were separated, divorced, or widowed. Common disability types of the participants include physical disability (40%), speech disability (9%), visual disability (12%), intellectual disability (12%), and individuals with multiple types of disabilities (15%).

**Table 1:** Weighted characteristics of the 5000 quantitative study participants

Background characteristics	Percentage (%)	Frequency (n)
Gender		
Female	49.1	2455
Male	50.9	2545
Age in years		
10–19	29.8	1490
20–29	22.9	1145
30–39	23.1	1155
40–49	14.9	745
50–59	9.2	460
Access to formal education		
Had access	51.5	2575
No access	48.5	2425
Education		
No formal education	48.5	2425
Primary education	28.4	1420
Secondary education	17.4	870
Post-secondary education	5.7	285
Residence		
Urban	16.3	815
Rural	83.7	4185
Household wealth quintile		
Poorest	20.0	1000
Poorer	20.1	1005
Middle class	19.9	995
Wealthy	20.1	1005
Wealthiest	20.0	1000
Marriage		
Married	37.4	1870
Unmarried	54.1	2705
Separated	2.6	130
Divorced	4.6	230
Widowed	1.4	70
Type of disability		
Physical disability	40.2	2010
Hearing disability	2.1	105
Speech disability	9.0	450
Visual disability	11.6	580
Intellectual disability	12.0	600
Autism or autism spectrum disorders	2.6	130
Cerebral palsy	4.1	205
Multiple disability	14.6	730
Down syndrome	0.5	25
Mental illness leading to disability	2.5	125
Deaf-blindness	0.3	15
Other disability	0.5	25

Table 2 shows the characteristics of the 15 participants interviewed in the qualitative phase of the study. Of the 15 qualitative participants, 8 were male and 7 were female. Three of them were from the age group 20 to 29 years, 7 were from the age group 30 to 39 years, 2 were from the age group 40 to 49 years, and 3 were from the age group 50 to 59 years. Four of them did not receive any formal education, 6 completed primary level education, 4 completed secondary level education, and only one completed post-secondary level education. The majority (12 out of 15) of qualitative participants were residents of rural areas. All of them were married, though 2 were divorced and 1 was separated at the time of qualitative data collection. Nine of them had physical disability, 3 had visual disability, one had hearing impairment, one had speech impairment, and one had cerebral palsy.

**Table 2:** Characteristics of the 15 qualitative study participants

Background characteristics	Number
Gender	
Female	7
Male	8
Age	
10–19	0
20–29	3
30–39	7
40–49	2
50–59	3
Access to formal education	
Had access	11
No access	4
Education	
No formal education	4
Primary education	6
Secondary education	4
Post-secondary education	1
Residence	
Urban	3
Rural	12
Household wealth quintile	
Poorest	6
Poorer	7
Middle class	1
Wealthy	1
Wealthiest	0
Marriage	
Married	12
Separated	1
Divorced	2
Type of disability	
Physical disability	9
Hearing disability	1
Speech disability	1
Visual disability	3
Cerebral palsy	1

### Pregnancy service use

#### Antenatal care service utilization

**Fig. 1:**
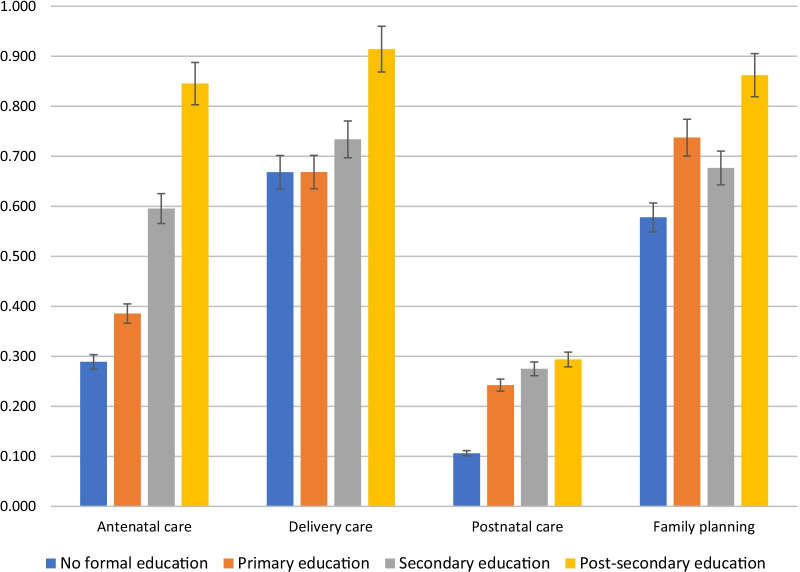
Pregnancy and family planning service use vs. education level of the participants. All survey participants were characterized as having no formal education, primary education, secondary education, or post-secondary education. For all 590 mothers with disabilities surveyed, the proportion using antenatal care, delivery care, and postnatal care is reported for each level of education. For all 1954 persons with disabilities (1239 male and 715 female) who responded to the survey’s family planning method questions, the proportion using family planning is reported for each level of education

Antenatal care utilization data was collected from all 590 female survey participants who had been pregnant during their lifetime. The proportion of female participants who utilized antenatal care services during pregnancy is 28.9% for non-formally educated mothers, 38.6% for mothers that attended primary school, 59.5% for mothers that attended secondary school, and 84.5% for mothers with post-secondary education (Fig. 1). The chi-squared test between education level and antenatal care use is statistically significant, with *P* < 0.001.

Univariate logistic regression results examining the effect of education on antenatal care use is presented in Table 3. The findings suggest that the odds of using antenatal care for a disabled woman who attended primary school is 1.7 times than for a disabled woman with no formal education (OR = 1.70; 95% CI 1.12, 2.56). For a disabled woman who attended secondary school and completed post-secondary education, their odds of using antenatal care is 3.4 times (OR = 3.44; 95% CI 2.19, 5.39) and 13.0 times (OR = 13.02; 95% CI 4.77, 35.53) than a non-formally educated woman, respectively (Table 3).

**Table 3:** Logistic regression models for education on antenatal, delivery, and postnatal care utilization

	Antenatal care				Delivery care				Postnatal care			
	Model 1		Model 2		Model 1		Model 2		Model 1		Model 2	
	Odds ratio		Odds ratio		Odds ratio		Odds ratio		Odds ratio		Odds ratio	
Education												
Primary education (vs. No formal education)	1.695	(1.124–2.556)	1.268	(0.816–1.969)	1.273	(0.848–1.913)	1.047	(0.679–1.614)	1.478	(0.881–2.477)	1.045	(0.597–1.828)
Secondary education (vs. No formal education)	3.439	(2.193–5.392)	1.979	(1.195–3.277)	2.256	(1.364–3.731)	1.467	(0.842–2.559)	2.650	(1.568–4.478)	1.369	(0.748–2.507)
Post-secondary education (vs. No formal education)	13.018	(4.770–35.529)	6.212	(2.153–17.925)	3.629	(1.224–10.761)	1.799	(0.569–5.688)	3.792	(1.649–8.723)	1.316	(0.514–3.366)
Residence												
Urban (vs. Rural)			1.079	(0.733–1.587)			0.898	(0.606–1.331)			1.070	(0.674–1.699)
Household wealth quintile												
Poor (vs. Poorest)			1.334	(0.790–2.254)			1.225	(0.736–2.038)			0.746	(0.380–1.466)
Medium (vs. Poorest)			1.298	(0.731–2.306)			0.994	(0.570–1.735)			0.813	(0.396–1.672)
Wealthy (vs. Poorest)			1.259	(0.706–2.245)			1.330	(0.751–2.356)			1.142	(0.576–2.266)
Wealthiest (vs. Poorest)			2.346	(1.262–4.362)			2.584	(1.289–5.179)			2.609	(1.311–5.192)
			0.938	(0.914–0.962)			0.955	(0.930–0.981)			0.929	(0.902–0.958)

Model 1 is the unadjusted logistic regression for education on pregnancy service (antenatal care, delivery care, and postnatal care) use. Model 2 is the logistic regression for education on pregnancy service use adjusting for type of residence, wealth quintile, and age. Values of 95% confidence intervals are given in brackets. The analysis was performed on all 590 Bangladeshi mothers with disabilities surveyed in this study

In the multivariable logistic regression model adjusting for area of residence, wealth, and age (Table 3), the adjusted odds of using antenatal care for a disabled woman who attended primary school is 1.3 times (AOR = 1.27; 95% CI 0.82, 1.97) than for a disabled woman with no formal education. For a disabled woman who attended secondary school and completed post-secondary education, their adjusted odds of using antenatal care is 2.0 times (AOR = 1.98; 95% CI 1.20, 3.28) and 6.2 times (AOR = 6.21; 95% CI 2.15, 17.93) than a non-formally educated woman, respectively (Table 3).

Qualitative findings suggest that participants’ educational attainment has a profound effect on their antenatal care service utilization. Chemon, a 40-year-old woman with visual impairment and higher educational attainment, shared that she sought medical treatment several times during her pregnancy.

*I did ultrasonography many times. I had too many difficulties! I couldn’t lift my head. I felt light headed and I vomited too much. At that time, I could not walk and move around at all. I couldn’t do anything. I couldn’t do any chores at home either. As I had too much trouble, I went to the doctors even twice or thrice in a month.*

*All of my family members are educated. That’s why they supported me a lot in my pregnancy. Also I am an educated girl, my educational background helped me to be sensible about these issues (reproductive health).*

(Chemon, 40-year-old woman, visual impairment, class 9 passed, married, middle class, urban area)

Chemon consulted with doctors during her pregnancy, receiving treatment at *Shurjer Hashi Clinic* and also meeting with another doctor to corroborate her health condition. It shows that she was more conscious about her pregnancy, actively seeking out consultation from more than one doctor to avoid complications. At the end, she faced no delivery complications such as preterm pain and anemia. According to Chemon, she belongs to an educated family, and they were all accommodating toward her pregnancy. Her educational background may have played a positive role in triggering her to be more conscious about her health conditions, leading her to seek antenatal care services.

Qualitative findings also suggest that when persons with disabilities try to acquire services from healthcare providers, those with limited formal education are often stigmatized and discriminated against. Rita, a 35-year-old woman with a physical disability and lower educational attainment, shared that she went to seek services during her pregnancy but was shamed with negligence due to her limited formal education and lack of understanding on maternal and child health (MNCH) issues.

*I went to the hospital to check my baby’s condition....there was a girl who checked...asked me my pregnancy duration...I said I don’t know...She asked, ‘why did you get pregnant then? Why did you sleep with the guy (husband)?’ I was very upset then. She shouted at me, as I do not understand these pregnancy things and I am not educated like them.*

(Rita, 35-year-old woman, physical disability, class 4 passed, married, poor, rural area)

Rita experienced discrimination while seeking services, due to her lack of MNCH knowledge stemming from lower educational attainment. These kinds of disrespectful attitudes by healthcare providers are not appropriate; however Rita would not have experienced such misbehavior if she had knowledge about her pregnancy duration.

It can be strongly reasoned, from the qualitative findings, that educational attainment positively contributes to improving antenatal care service utilization. Asma, a 30-year-old woman with a physical disability and holder of a master’s degree in English, shared that she sought pregnancy services even in the midst of the COVID-19 pandemic. When the country was in lockdown due to the pandemic, healthcare services were running on a limited scale and physician availability had decreased. People were also afraid to go to the hospital, in fear of getting infected. However, even at that time, Asma sought healthcare services.

*In the COVID-19 lockdown situation, hospitals are open with very few employees and most of the doctors are not available. I tried several times to get an appointment with a gynecologist. In the first hospital, we were informed that there are no doctors available. Then we tried for another doctor and luckily there we got the chance to consult with one doctor…Though I am from a lower middle class family and had to starve in this lockdown situation often, my family prioritized my health condition. I made sure my family understood the seriousness of my pregnancy condition. My education helped me here to be conscious about these issues (SRH).*

(Asma, 30-year-old woman, physical disability, masters passed, married, lower-middle class, urban area)

Asma elaborated that due to the COVID-19 pandemic, her family was facing economic constraints like many others, but they prioritized her pregnancy condition and acquired professional help. According to Asma, her family’s support and her self-consciousness helped her through these difficulties, while her educational attainment also worked in favor of her SRH understanding.

#### Delivery care service utilization

Delivery care utilization data was collected from all 590 female survey participants who had been pregnant during their lifetime. The proportion of female participants who utilized delivery care services is 66.8% for non-formally educated mothers, 66.9% for mothers who attended primary school, 73.4% for mothers who attended secondary school, and 91.4% for mothers with post-secondary education (Fig. 1). The chi-squared test between education level and delivery care use is statistically significant, resulting in *P* < 0.05.

Univariate logistic regression results examining the effect of education on delivery care use is presented in Table 3. The findings show that the odds of using delivery care for a disabled mother who attended primary school is 1.3 times (OR = 1.27; 95% CI 0.85, 1.91) than for a disabled mother with no formal education. For a disabled mother who attended secondary school and completed post-secondary education, their odds of using delivery care is 2.3 times (OR = 2.26; 95% CI 1.36, 3.73) and 3.6 times (OR = 3.63; 95% CI 1.22, 10.76) than a non-formally educated mother, respectively (Table 3).

In the multivariable logistic regression model of delivery care use adjusting for area of residence, wealth, and age (Table 3), the adjusted odds of delivery care usage for a disabled mother who attended primary school, secondary school, and completed post-secondary education is 1.0 (AOR = 1.05; 95% CI 0.68, 1.61), 1.5 (AOR = 1.47; 95% CI 0.84, 2.56), and 1.8 times (AOR = 1.80; 95% CI 0.57, 5.69) than delivery care usage for a disabled mother with no formal education, respectively (Table 3).

In the case of delivery care service utilization, most qualitative participants (10 out of 15) reported that they prefer home delivery care through a birth attendant. This is true for both the educated and uneducated participants. Yet educated participants, who had certain health issues like gestational diabetes, preterm pain or labor, anemia, or breech position during pregnancy, were found to seek more healthcare services than the uneducated participants who faced the same difficulties. Lack of education negatively influences persons with disabilities’ formal delivery care service utilization. Rita, a 35-year-old woman with a physical disability and lower educational attainment, shared that she suffered from several health complications during delivery, but did not go to the hospital or seek any formal healthcare services.

*I got sick when I was pregnant with my last child….Everyone told me to go the hospital. During delivery, the placenta tore. I was about to die. It bled intensively; it could be measured to a bucket, even more than a bucket! I fainted. One of my neighbors went to the nearest pharmacy and called the ‘doctor brother’ (drug-seller). Then he gave me saline and my condition got better… My husband did not take me to the hospital. He is not interested to go to the hospital for this womanly (child delivery) issue. He does not allow us (family members) to go to the hospital for any other treatments also…. They (in-law’s family members) prefer ‘kabiraj’ (traditional healer) in case of any sickness.*

(Rita, 35-year-old woman, physical disability, class 4 passed, married, poor, rural area)

Rita experienced a difficult delivery but declined formal healthcare services due to a lack of knowledge and financial constraints. Her husband opted for a home delivery and did not prefer any kind of medical treatment regarding Rita’s pregnancy and maternal health issues, due to his belief in ‘kabiraji chikitsa’ (traditional healing method). Rita’s husband had a strong gender influence on her, controlling her formal health care service-seeking ability. Beside these, Rita’s educational qualification is below primary education (class 4) and her husband had no formal education, which led them to fail to secure a basic livelihood. Though poverty is one of the important factors here, the couple’s lack of SRH knowledge due to lower educational attainment led them to avoid formal healthcare services. They could not afford delivery care services, but also did not desire them because of their limited education and cultural belief in the traditional healing method.

Besides educational attainment, economic status was found to be a major factor which negatively contributes to participants’ SRH service utilization. Participants with lower economic backgrounds shared that hospital services are often too costly and unaffordable for them. A 40-year-old physically disabled woman with no formal education from a rural area complained about her economic backdrop while facing delivery complications; she lost her child during delivery.

#### Postnatal care service utilization

Postnatal care service utilization data was collected from all 590 female survey participants who had been pregnant during their lifetime. The proportion of female participants who utilized postnatal care services is 10.6% for non-formally educated mothers, 24.2% for mothers who attended primary school, 27.5% for mothers who attended secondary school, and 29.4% for mothers with post-secondary education (Fig. 1). The chi-squared test between education level and postnatal care use is statistically significant, resulting in *P* < 0.001.

Univariate logistic regression results examining the effect of education on postnatal care use is presented in Table 3. The model shows that the odds of using postnatal care for a disabled mother who attended primary school is 1.5 times than using postnatal care for a disabled mother with no formal education (OR = 1.48; 95% CI 0.88, 2.48). For a disabled mother who attended secondary school and completed post-secondary education, their odds of using postnatal care is 2.7 times (OR = 2.65; 95% CI 1.57, 4.48) and 3.8 times (OR = 3.79; 95% CI 1.65, 8.72) than a non-formally educated mother, respectively (Table 3).

In the multivariable logistic regression model of postnatal care use adjusting for area of residence, wealth, and age (Table 3), the adjusted odds of postnatal care usage for a disabled mother who attended primary school, secondary school, and completed post-secondary education is 1.0 (AOR = 1.05; 95% CI 0.60, 1.83), 1.4 (AOR = 1.37; 95% CI 0.75, 2.51), and 1.3 times (AOR = 1.32; 95% CI 0.51, 3.37) than postnatal care usage for a disabled mother with no formal education, respectively (Table 3).

Qualitative data reveals that highly educated participants seek more postnatal healthcare services. Chemon, a 40-year-old woman with visual impairment and higher educational attainment, visited healthcare facilities several times with her children after pregnancy.

*I visited the nearest health complex, several times after my pregnancy. I used to go there for my children’s vaccination or getting vitamin tablets. I also went there for my after pregnancy weakness (less energetic and dizziness) treatment. My husband and family members are educated and they were very supportive in these issues.*

(Chemon, 40-year-old woman, visual impairment, class 9 passed, married, middle class, urban area)

Chemon received postnatal care treatment after her delivery, and her family was very supportive of this. As Chemon belongs to an educated family, she faced fewer difficulties in accessing SRH services and received support that fulfilled her needs.

On the other hand, some lower educated study participants reported that they do not think it is important to seek postnatal care services, as they believe it will not bring any health benefits. Rita, who only passed class 4, shared she never utilized professional healthcare services for postnatal care, including child vaccination.

*I never went to hospital after giving birth, even for my children’s vaccination! Many people go with their children for vaccination. But I never went. Nowadays, my daughters want to go to hospital with their children, but I do not want them to go. What is the benefit of going there? Nothing!*

(Rita, 35-year-old woman, physical disability, class 4 passed, married, poor, rural area)

Rita found postnatal care services unreasonable and never sought this type of service. She even discouraged her daughters from vaccinating their children. Rita’s educational background led her to stigmatize formal SRH services. Her husband’s traditional cultural views and their poverty, as mentioned in her previous quote, adds to their reluctance in seeking these services.

### Family planning service utilization

Family planning service utilization data was collected from 1954 male and female survey participants. Among all the participants who do not have any formal education, 57.8% of them used family planning methods during their lifetime (Fig. 1). However, participants who exhibit more than 10 years of formal schooling have 86.2% of family planning method usage. Those who attended only primary or secondary school have proportions of family planning method usage at 73.7% and 67.7%, respectively. The chi-squared test between education level and family planning use is statistically significant, with *P* < 0.001.

Univariate logistic regression results examining the effect of education on family planning method use is presented in Table 4. The model shows that the odds of using family planning methods for a disabled individual who attended primary school is 2.0 times than using family planning methods for a disabled individual with no formal education (OR = 2.03; 95% CI 1.62, 2.54). For a disabled individual who attended secondary school and completed post-secondary education, their odds of using family planning methods is 1.7 times (OR = 1.71; 95% CI 1.33, 2.19) and 3.4 times (OR = 3.36; 95% CI 1.97, 5.73) than a non-formally educated individual, respectively (Table 4).

**Table 4:** Logistic regression models for education on family planning utilization

	Family planning			
	Model 1		Model 2	
	Odds ratio		Odds ratio	
Education				
Primary education (vs. no formal education)	2.029	(1.619–2.542)	2.088	(1.652–2.639)
Secondary education (vs. no formal education)	1.709	(1.333–2.192)	1.719	(1.317–2.245)
Post-secondary education (vs. no formal education)	3.361	(1.973–5.726)	3.523	(2.022–6.138)
Residence				
Urban (vs. rural)			0.795	(0.647–0.976)
Household wealth quintile				
Poor (vs. poorest)			1.369	(1.04–1.801)
Medium (vs. poorest)			1.467	(1.093–1.969)
Wealthy (vs. poorest)			1.335	(0.985–1.809)
Wealthiest (vs. poorest)			1.360	(0.973–1.902)
Age			1.008	(0.999–1.018)

Model 1 is the unadjusted logistic regression for education on family planning use. Model 2 is the logistic regression for education on family planning use adjusting for type of residence, wealth quintile, and age. Values of 95% confidence intervals are given in brackets. The analysis was performed on all 1954 persons with disabilities (1239 male and 715 female) in Bangladesh who responded to the survey’s family planning questions

In the multivariable logistic regression model adjusting for area of residence, wealth, and age (Table 4), the adjusted odds of using family planning methods for a disabled individual who attended primary school is 2.1 times than using family planning methods for a disabled individual with no formal education (AOR = 2.09; 95% CI 1.65, 2.64). For a disabled individual who attended secondary school and completed post-secondary education, their adjusted odds of using family planning methods is 1.7 times (AOR = 1.72; 95% CI 1.32, 2.25) and 3.5 times (AOR = 3.52; 95% CI 2.02, 6.14) than a non-formally educated individual, respectively. Education level increased disabled individuals’ adjusted odds of using family planning methods more than area of residence, wealth, or age (Table 4).

Qualitative findings show that education positively contributes to family planning service utilization among the study participants. Participants’ preference to use family planning methods increased with their educational attainment. A 52-year-old educated man with visual impairment shared that he and his wife were well aware of family planning methods.

*In the first 2–3 years of my marriage, I did use condom: Raja condom (brand name). After the birth of my daughter, my wife started taking birth control pills….I learned these from my friends. As I am an educated man I got chances to learn from radio, television, roadside advertisements, leaflets, etc.*

(Rahim, 52-year-old man, visual impairment, H.S.C. passed, married, lower middle class, rural area)

This couple used different family planning methods during their conjugal life. According to Rahim, his education helped him to learn about family planning methods from different media sources.

A closer look at the qualitative findings suggests that participants who have comparatively lower educational attainment were less aware of family planning methods, and hence suffer the most from SRH related issues. Banu, a 35-year-old woman with a physical disability and no formal education, shared that she was only 12 when she married. She got pregnant from her first intercourse, and she did not know about family planning methods then.

*I got married at the age of 12. After my first menstruation I got married and started living with my husband. After that my menstruation got stopped…. When I did not get my period for 4–5 months, then it was noticed. My mother asked me if I’m getting my period. I said that I had not been getting my period for 3–4 months. Then my mother told my grandmother about my pregnancy….I was not clever enough about that like today’s school going girls. I was unaware about it.*

(Banu, 35-year-old woman, physical disability, no formal education, married, poor, rural area)

Banu was completely unaware of any family planning methods and experienced an unintentional pregnancy. She learned of her pregnancy when her family members noticed her physical appearance or menstrual irregularity. Though marriage at an early age was one of the reasons for her early pregnancy, she mentioned that she lacked awareness of her pregnancy due to lower educational attainment.

Banu also commented on her husband’s ignorance toward family planning methods.

*What will he think about it (family planning)! Where’s his educational background for it*!

(Banu, 35-year-old woman, physical disability, no formal education, married, poor, rural area)

She was doubtful about her husband’s knowledge regarding family planning methods, as his education did not suffice on this topic. Banu values education and realized that her husband’s lack of education is mainly responsible for his ignorance.

Chang, a 35-year-old woman with a physical disability and no formal education, shared that she did not have the chance to educate herself on family planning, as her household was too poor to provide her with educational support. She belongs to an indigenous community in a remote hilly area. Chang was married at the age of 12 and had no idea about family planning methods; she experienced miscarriage during her first pregnancy.

*I knew that I was pregnant. But I couldn’t tell it to the doctor, as I was shy! I took the medicine (skin care) as I didn’t know that medicine is prohibited. Then the baby was gone… I don’t know anything about these things women take nowadays (family planning methods)….*

(Chang, 35-year-old woman, physical disability, no formal education, divorced, poor, rural area)

Chang clearly experienced a miscarriage due to her knowledge gap on family planning; she took a skin care medication which resulted in her miscarriage. Getting married at a young age and being an indigenous woman from a remote hilly area affected her educational attainment and communication skills, which affected her SRH service use. She failed to obtain family planning or pregnancy knowledge and services, which lead to her miscarriage.

Qualitative study participants sometimes harbored misconceptions from community members regarding family planning methods, spread by those with less education. Banu shared that she was taking injections as a family planning method for many years. Her neighbors tried to influence her not to use injections, as they believed it may harm her.

*They (neighbors) said, ‘Why do you use injections? You’ll be crippled. You already have disability, you’ll be further disabled. What will you do then? Your blood will turn to water. Now you can at least walk. If you keep using injections, you wouldn’t even be able to walk. You’ll get anemia.’*

(Banu, 35-year-old woman, physical disability, no formal education, married, poor, rural area)

Lack of knowledge on family planning methods, such as in the case of Banu’s neighbors, can affect individuals’ utilization of family planning methods. Different conceptions or misconceptions regarding family planning methods in one’s community or society is a determinant of individual opinions surrounding family planning methods. Misconceptions are deeply connected with an individual’s SRH understanding and overall educational attainment. Banu’s neighbors tried to confuse her with their limited knowledge to discourage her use of family planning methods. One may easily be influenced by these misconceptions and derailed from bettering their SRH if their educational background is not strong enough to push back against it.

## Discussion

Our study findings reveal that education greatly impacts participants’ SRH service use. As shown by quantitative analyses, education positively contributes in almost all cases of SRH service utilization: antenatal care, delivery care, postnatal care, and family planning. For these, the proportion of the service use generally increases as education level increases. Additionally, multivariable logistic regression findings adjusted for important covariates such as age and wealth quintile show that the odds of antenatal care, delivery care, postnatal care, and family planning use tend to increase with greater education levels. Similarly, qualitative findings show that educated participants are more inclined to utilize formal SRH services. On the other hand, participants with lower educational backgrounds rarely utilized formal SRH services and often experienced discrimination and mistreatment from healthcare providers because of a lack of knowledge on SRH issues.

In this study, the most used pregnancy service is delivery care, followed by antenatal care, then postnatal care. Out of these services, higher education leads antenatal care service use to increase the most. When considering pregnancy services, logistic regression findings show that formal education is key. Accounting for confounding variables, the multivariable logistic regression model displays that primary education is not enough for a disabled woman to have a marked increase in use of antenatal care. However, for a disabled woman who attended secondary school and completed post-secondary education, their adjusted odds of using antenatal care are 2 times and 6 times the odds of a non-formally educated woman, respectively. For delivery care and postnatal care, the models also demonstrate that primary education is not enough to improve SRH service use, and that secondary or post-secondary education may be required. Other studies also have odds ratios showing that education improves pregnancy service utilization for women with disabilities in LMICs [21–23]. In this study, qualitative analyses explain that higher educated participants prioritized obtaining professional pregnancy services, even when financially constrained, while lower educated participants tended to be misinformed and distrustful of pregnancy services. A scoping review cites limited reproduction-related education as an important theme barring women with disabilities in LMICs from accessing proper SRH services [24], as it prevents these women from factually understanding the benefits of these services.

The study findings also suggest that formal education improves the proportion of family planning method use in a statistically significant way. Primary education and secondary education show similar increased adjusted odds for family planning use, while post-secondary education shows the greatest increase in adjusted odds for family planning use. Other studies also assert that having greater education significantly increases the uptake of contraceptives for women with disabilities [25, 26]. A multivariate logistic regression model of reproductive-age women in Ethiopia showed similar trends in family planning use as that in this study [25]. According to qualitative analyses of this study, participants married at a very young age were found to have lower educational attainment and lower levels of family planning knowledge. They learned about family planning methods after their marriage, when they had already given birth to their children. Hence, increasing educational attainment for women with disabilities is essential in improving their family planning utilization. A study on contraception use among Ugandan women with disabilities suggests that education increases family planning use by enabling women to gain self-efficacy and overcome numerous systematic barriers like women’s subordination or family size norms [26].

As shown by the study findings, participants with poor knowledge on pregnancy and SRH issues often face discrimination and rude behavior from healthcare providers. This, coupled with a range of social, attitudinal, and physical barriers often create difficulties for them to access reproductive healthcare services [27]. Findings from previous studies in LMICs show that women with disabilities face greater challenges in accessing SRH services compared to women without disabilities [10, 11]. Furthermore, a systematic review on SRH for persons with disabilities in LMICs could not identify any interventions intended to promote persons with disabilities’ maternal health or family planning [11]. In order to improve maternal care services for women with disabilities, it is important to mainstream disability issues in the national training and education curriculum of all types of healthcare providers and enhance providers’ skills on respectful maternity care [28]. It is also important to educate persons with disabilities on different SRH issues and make them aware of SRH service availability.

In qualitative analysis, the study also finds participants' service seeking behavior and service utilization is strongly influenced by gender norms in the society. Women with disabilities often cannot decide for themselves when and where to go for SHR services. Like other South Asian societies, Bangladesh is mostly patriarchal and strictly follows gender norms; women are expected to be dependent and controlled by men throughout their lives, from labor market access to sexuality and reproductive choices [29]. Persons with disabilities are considered as an exception to sexual norms in society and get excluded further, where women with disabilities are seen as individuals who cannot decide for themselves and do not have sexual agency [30].

Recently, some national and international NGOs are offering promising youth-friendly services related to adolescent SRH including face-to-face counseling, social media campaigns, social gatherings, and clinical services [31]. These services complement government-sponsored efforts to bring adolescent health education into schools, and both are integral in implementing comprehensive sexuality education (CSE) for Bangladeshi youth. CSE covers gender equality, SRH, HIV, sexual rights, and freedom from violence while addressing the stigma associated with SRH in Bangladesh; hence, persons with disabilities need to be able to access these services for improved SRH outcomes.

Overall, efforts to formally educate persons with disabilities may positively contribute to better SRH service use and outcomes. This study reveals that 48.5% of the study participants have no access to formal education. Low education rates are a major barrier to persons with disabilities’ access to SRH information and services [5, 16, 24, 27, 32]. In LMICs, persons with disabilities who have never participated in formal education or dropped out of primary education have limited or no access to CSE through formal education [24]. Formal education is a crucial avenue in which youth can gain access to SRH education. For example, CSE taught to children with disabilities showed promising outcomes in South African schools [33]. This study recommends that future policy and action plans should involve increasing formal education as well as comprehensive sexual education for persons with disabilities.

There are several limitations to this study. The study adopted a cross-sectional design, which cannot draw causal relationships between the SRH service utilization and outcomes and educational attainment of the participants. However, a mixed-methods study design was adopted, which allowed us to comprehensively conclude that for persons with disabilities education positively contributes to SRH service utilization. Because SRH is a sensitive topic, some participants may not have shared their full SRH experiences. We could not explore the SRH sufferings and service utilization of participants with neurodevelopmental disorders and speech impairment directly from them, because of communication barriers. In these cases where the research team faced difficulties interviewing persons with disabilities, caregivers were interviewed instead. Only ever-married women with disabilities were included in our analysis, which does not account for the experiences of women with disabilities who were never married. Additionally, this study uses a representative sample for surveying the population and for choosing qualitative interviewees, which does not encapsulate the SRH experiences of every disabled citizen in Bangladesh.

## Conclusions

To our knowledge, this study is the first conducted in Bangladesh on the influence of education on SRH service utilization for persons with disabilities. We found that formal education positively contributes to better SRH service use for persons with disabilities, which may result in better health outcomes overall. The result of this study can be helpful for policymakers in designing effective interventions and programs for persons with disabilities to eventually lessen the SRH sufferings of this group. Ultimately, the government should make it mandatory to teach SRH to persons with disabilities, and current government programs to improve SRH for persons of disabilities need to be strengthened and sustained. Teachers should be educated about SRH and tasked to deliver comprehensive sexuality education through the hundreds of specialist schools, vocational institutions, and NGOs serving persons with disabilities. Lastly, more research is required on exploring the relationships between education and other SRH outcomes of persons with disabilities in LMICs, so that efforts to improve the SRH of persons with disabilities will be backed by well-informed data and evidence. This way, Bangladesh can meet the United Nations sustainable development goals (SDGs) related to SRH, and persons with disabilities can fully enjoy their SRH rights.

## Data Availability

The dataset supporting the conclusions of this article is available from the corresponding author upon reasonable request.

## Abbreviations

WHO: World Health Organization
SRH: Sexual and reproductive health
LMICs: Low and middle-income countries
NGO: Non-governmental organization
DPO: Disabled persons’ organization
STD: Sexually transmitted disease
MNCH: Maternal and child health
CSE: Comprehensive sexual education
SDGs: Sustainable development goals
